# *Kampo* Diagnostic Procedure, *Fuku shin*, Could Be a Useful Diagnostic Tool for Psychopathological Patients Suffering from Chronic Pain

**DOI:** 10.1155/2013/816216

**Published:** 2013-06-12

**Authors:** Young-Chang P. Arai, Makoto Nishihara, Shinsuke Inoue, Izumi Makino

**Affiliations:** Multidisciplinary Pain Center, Aichi Medical University, 21 Karimata Nagakutecho, Aichigun, Aichi 480-1195, Japan

## Abstract

*Kampo*, or traditional Japanese herbal medicine, has been used for clinical practice in Japan. The most appropriate *Kampo formula* should be chosen for each individual based on specific diagnostic procedures. *Fuku shin*, the abdominal exam, is one of the most important diagnostic procedures. There are several *Fukusho*s (abdominal conformations). Depression and anxiety have been shown to be related to *Shin ka hi koh*, epigastric obstructive hardness, and neuroses have an association with *Kyoh kyoh ku man*, hypochondriac distress. The aim of our study was to compare *Fukusho*s at each level of depression and anxiety symptoms and to assess the associations between occurrence and pain catastrophizing. Two hundred and twenty-nine patients were assigned to high-, moderate-, or low-level anxiety-depression groups based on the hospital anxiety and depression scale and were investigated for occurrence of major *Fukusho*s. Moreover, the associations between occurrence and the pain catastrophizing scale (PCS) were analyzed. The moderate and high anxiety-depression groups showed a higher occurrence of *Shin ka hi koh* [low, 29%; moderate, 51%; high, 67%] (*P* < 0.0001). In contrast, the relationship between the occurrence of *Kyoh kyoh ku man* and the PCS showed a significant albeit small correlation (*r*_*s*_ = 0.1296, *n* = 229, *P* = 0.0491).

## 1. Introduction

*Kampo*, or traditional Japanese herbal medicine based on traditional Chinese herbal medicine, has been used for the treatment of chronic pain in Japan [1–3]. Traditional Chinese medicine and *Kampo* developed a characteristic system [1–3]. There are essentially three dichotomies and three substance concepts in the system. The three dichotomies are Yin-You (ying-yang), Kyo-Jitsu, and Netsu-Kan. In English, they could be translated as positive-negative, hollow-full, and hot-cold, respectively. The three substance categories are Ki (Qi), Ketsu and Sui. Ki (Qi) is energy fundamental to living things. In contrast to the Ki (Qi) concept, Ketsu, and Sui are material and much closer to the usual concepts of blood and body fluids, respectively. In *Kampo*, the healthy state of human beings means a well-balanced or undeviated condition of the three dichotomies and the three substance concepts. Disease due to deviation or imbalance is assessed by four specific diagnostic procedures. According to *Kampo theory*, the most appropriate *Kampo formula* should be chosen for each individual based on four diagnostic procedures [1–4]. *Fuku shin*, the abdominal exam, is unique to Japan and is one of the most important approaches of the four diagnostic procedures in *Kampo* [2, 3, 5]. There are several *Fukusho*s (abdominal conformations) when administering *Fuku shin*. There are four major* Fukusho*s: *Shin ka hi koh*, epigastric obstructive hardness; *Kyoh kyoh ku man*, hypochondriac distress and fullness; *Ri kyuh*, inside spasm (rectus muscle tension); *Sho fuku koh man*, lower-abdomen hardness and fullness [2, 3]. In* Kampo*, depression and anxiety have been shown to be related to *Shin ka hi koh*, epigastric obstructive hardness, and neuroses have an association with *Kyoh kyoh ku man*, hypochondriac distress [2, 3].

Recent studies have shown the role of psychological variables on patients' pain experience and quality of life. Catastrophizing, anxiety, and depression are major factors [6]. However, one of the important issues for clinicians is the choice of screening tool. The hospital anxiety and depression scale (HADS) has been used in a number of studies [6]. The HADS was designed to assess two separate dimensions of anxiety and depression. The HADS consists of 14 items; the anxiety (HADS-A) and depression (HADS-D) subscales each include 7 items [6, 7]. A 4-point response scale (from 0 representing absence of symptoms to 3 representing maximum symptoms) is used, with possible scores for each subscale ranging from 0 to 21. Moreover, catastrophizing has been assessed by the pain catastrophizing scale (PCS) [8–10]. The PCS is a 13-item scale that assesses three types of negative thinking styles related to pain. Subjects are asked to reflect on past painful experiences and indicate on a 5-point scale ranging from 0 (“not at all”) to 4 (“always”) the degree to which they experienced each of the 13 thoughts or feelings when in pain [8–10].

We hypothesized that *Shin ka hi koh*, epigastric obstructive hardness, and *Kyoh kyoh ku man*, hypochondriac distress, are signs of psychiatric comorbidity. We thus compared the occurrence of four major *Fukusho*s at each level of depression and anxiety symptoms to assess the associations between occurrence and level of depression-anxiety symptoms or pain catastrophizing.

## 2. Methods

Retrospective analysis from April 2011 to November 2012 was performed on 842 patients suffering from chronic pain who visited the pain center of Aichi Medical University Hospital. All patients were referred from other hospitals to the pain center. Patients who underwent *Kampo diagnosis* for *Kampo formula* were included.

The HADS and the PCS are routinely administered to all patients on admission to the pain center. The existing translation of the Japanese version of HADS and PCS [7, 11] is used in our daily clinical practice. The HADS, the PCS, and the abdominal exam records of *Kampo diagnosis* from April 2011 to November 2012 were extracted from medical records for the present study, after receiving approval from the Ethics Committee of Aichi Medical University. Consequently, 229 subjects were included in the present study.

In the abdominal exam records, we focused on and investigated the occurrence of only four major *Fukusho*s out of a wider group of documented abdominal findings: *Shin ka hi koh*, epigastric obstructive hardness; *Kyoh kyoh ku man*, hypochondriac distress and fullness; *Ri kyuh*, inside spasm (rectus muscle tension); *Sho fuku koh man*, lower-abdomen hardness and fullness [2, 3].

*Shin ka hi koh*, epigastric obstructive hardness: patients report that their epigastrium feels “stuffed,” and upon palpation by the physician, tightness and resistance are detected.

*Kyoh kyoh ku man*, hypochondriac distress: there is a feeling of fullness in the hypochondrium, as well as distress and pain. It can be verified objectively as resistance and pressure pain.

*Ri kyuh*, inside spasm (rectus muscle tension): upon palpation by the physician, a spasm is detected, which feels like a sudden jerk or contraction beneath the surface of the abdomen.

*Sho fuku koh man*, lower-abdomen hardness and fullness: the lower abdomen is inflated and shows resistance as well.

The patients were assigned to high-, moderate-, or low-level anxiety-depression groups based on the subscales of the HADS [12, 13]. To be in the high group, scores had to be high on both the depression and anxiety subscales (i.e., at least 9 on each, HADS total score ≥18). To be in the low group, scores had to be low on both subscales (total score ≤12), and the moderate group included all others not meeting high or low criteria.

Values are numbers or median [range]. Patient's characteristics were compared using one-way ANOVA followed by Tukey's test or chi-square test. The occurrence of each *Fukusho* was analyzed using chi-square test. Associations between the occurrence of each *Fukusho* and the level of depression-anxiety symptoms or pain catastrophizing were analyzed using Spearman's rank correlation coefficient (*r*_*s*_). A *P* value of < 0.05 was considered significant.

## 3. Results

Patient's characteristics are presented in Table 1. Seventy of the patients were in the low psychopathology group, 80 in the moderate psychopathology group, and 79 in the high psychopathology group. There were no significant differences between the patients' characteristics among the three groups. The occurrence of *Shin ka hi koh *was higher in the moderate and high anxiety-depression groups than in the low anxiety-depression group [low, 29%; moderate, 51%; high, 67%] (*P* < 0.0001) (Table 2). There were no significant differences in the occurrence of *Kyoh kyoh ku man, Ri kyuh*, and *Sho fuku koh man* among the three groups. Moreover, the relationship between the occurrence of *Shin ka hi koh* and level of depression and anxiety symptoms showed a significant and positive albeit small correlation (*r*_*s*_ = 0.3086, *n* = 229, *P* < 0.0001). In contrast, the relationship between the occurrence of *Kyoh kyoh ku man* and the PCS showed a significant and positive albeit small correlation (*r*_*s*_ = 0.1296, *n* = 229, *P* = 0.0491) (Table 3).

## 4. Discussion

The main findings of the present study are that while the moderate and high anxiety-depression groups showed a higher occurrence of *Shin ka hi koh*, epigastric obstructive hardness, the relationship between the occurrence of *Kyoh kyoh ku man* and the pain catastrophizing scale (PCS) showed a significant and positive albeit small correlation, based on one type of *Kampo diagnosis*, *Fuku shin* (the abdominal exam).

Recent studies have shown the role of psychological variables on patients' pain experiences and quality of life. Catastrophizing, anxiety, and depression are major factors [6]. The HADS was originally designed to assess two separate dimensions of anxiety and depression [6, 7]. As a brief screening tool, the HADS scale has increased in popularity. A review article concludes that the HADS performs well in screening for cases of anxiety disorders and depression in patients from nonpsychiatric hospital clinics [14]. Catastrophizing has been assessed by PCS [8–10]. Pain catastrophizing is an exaggerated, negative focus on pain and related to various indices of psychological distress. However, we have to keep in mind that such tests are by no means sophisticated or rigorous enough to establish anything other than a relatively superficial psychiatric diagnosis. We thus need further studies.

There are the four diagnostic procedures that make up what is called in *Kampo* the four exams by which *Kampo formula* is prescribed for each individual [1–4]. One is called *Setsu shin*, the tactile exam, which consists of *Fuku shin*, the abdominal exam, and *Myaku shin*, the pulse exam. In *Kampo*, sensations from the outside rule the pulse and internal damages govern the abdomen [1, 2]. This means that diagnosis of exogenous agent-induced disease such as acute febrile disease depends on the pulse, while the progress of chronic illness is taken to be endogenously induced and the diagnosis should be made in accordance with abdominal signs. That is, chronic illness is induced by mental factors to such an extent and an abdominal sign could indicate psychiatric illness. 

*Fuku shin* is the component of examination based on *Kampo* abdominal palpation. Physicians have the patient lie on his or her back with both legs extended. The physicians stand on the side of the patients, examine with the hands, and stroke from the chest to the abdomen to determine whether the abdominal wall is thick or thin and to sense the abdominal wall condition [2, 3]. *Fuku shin* contributes to the classification of pathology on a theoretical level, and furthermore it is first and foremost a practical, treatment-oriented diagnosis method.

When *Fukusho*, *Shin ka hi koh* (epigastric obstructive hardness), is present, the most commonly used recipes are specific *Kampo formulas*, *Hangekohbokutoh*, and *Kohsosan*. When *Kyoh kyoh ku man* (hypochondriac distress and fullness) is present, we prescribe *Shosaikotoh*, *Daisaikotoh*, and *Kamishoyousan*. These formulas can treat psychiatric illness [1–3]. In fact, when these *Fukusho*s were present, we prescribed these formulas for the patients. Furthermore, the present study showed that moderate and high anxiety-depression patients had *Shin ka hi koh *and there was a significant association between the occurrence of *Kyoh kyoh ku man* and the PCS. We thus postulate that *Fuku shin*, the abdominal exam, could be a diagnostic tool for psychiatric illness.

In conclusion, moderate and high anxiety-depression patients displayed a higher occurrence of the abdominal sign, *Shin ka hi koh* (epigastric obstructive hardness), and there was a significant association between the occurrence of *Kyoh kyoh ku man* and PCS.

## Figures and Tables

**Table 1:** Patient's characteristics.

	Low	Moderate	High	*P*
Sex (M/F)	20/50	18/62	24/55	0.5070
Age (years)	61 [15–86]	53 [13–86]	49 [23–86]	0.5301
Weight (kg)	52 [34–82]	52.5 [32–82]	51 [38–92]	0.2581

Values are numbers or median [range]. There were no significant differences.

**Table 2:** Occurrence of each abdominal conformation.

	Low	Moderate	High	*P*
*Shin ka hi* *koh* (+/−)	20/50 (29%)	41/39 (51%)*	53/26 (67%)*	<0.0001
*Kyoh kyoh ku man* (+/−)	20/50 (29%)	27/53 (34%)	26/53 (33%)	0.3764
*Ri kyuh* (+/−)	22/48 (31%)	17/63 (21%)	34/45 (29%)	0.0921
*Sho fuku koh man* (+/−)	41/29 (59%)	37/43 (46%)	42/37 (53%)	0.7934

Values are numbers. *Significantly different from low group.

**Table 3:** Associations between the occurrence of each abdominal conformation and the pain catastrophizing scale (Spearman's rank correlation coefficient (*r*_*s*_)).

	*Shin ka hi koh *	* Kyoh kyoh ku man *	*Ri kyuh *	*Sho fuku koh man *
*r*_*s*_	0.09387	0.1296	0.06221	0.02733
*P*	0.1568	**0.0491 **	0.3487	0.6807
